# Structural Characteristics, Comparative Analyses, and Conservation Significance of the Complete Chloroplast Genome of the Critically Endangered *Lithocarpus yongfuensis* (Fagaceae)

**DOI:** 10.1002/ece3.72833

**Published:** 2026-01-09

**Authors:** Fengzhi Gu, Lifang Yang

**Affiliations:** ^1^ School of Ecology and Environmental Science, State Key Laboratory of Vegetation Structure, Function and Construction (VegLab) Yunnan Key Laboratory of Biological Adaption, Conservation and Utilization, Yunnan University Kunming China; ^2^ Yunnan Key Laboratory for Integrative Conservation of Plant Species With Extremely Small Populations Kunming Yunnan China; ^3^ Asian Elephant Yunnan Field Scientific Observation and Research Station and Yunnan Asian Elephant Field Scientific Observation and Research Station of the Ministry of Education Yunnan University Kunming China

**Keywords:** chloroplast genome, comparative analyses, *Lithocarpus yongfuensis*, phylogenomics, positive selection, SSR analysis

## Abstract

This study aims to delineate the chloroplast (cp) genome of the critically endangered *Lithocarpus yongfuensis* (Fagaceae), with fewer than 10 wild individuals known. Genomic information for this species is scarce, hindering conservation strategies. We sequenced, assembled, and annotated its complete cp genome, analyzed its structure, and conducted comparative and phylogenetic analyses within the genus *Lithocarpus*. The cp genome is 161,258 bp in length, exhibiting a typical quadripartite structure and containing 131 annotated genes (86 protein‐coding, 8 rRNA, and 37 tRNA genes). Comparative analysis revealed a conserved genomic architecture across the genus, with two protein‐coding genes (*rpoC2* and *matK)* showing evidence of positive selection. The *rps16–trnK‐UUU* intergenic spacer was identified as a potential DNA barcode for distinguishing *L. yongfuensis*. Phylogenetic analysis based on complete cp genomes placed *L. yongfuensis* within the Southeast China clade, closely allied to *L. crassifolius* , *L. litseifolius*, and *L. brevicaudatus* . These findings provide essential genomic resources for conservation genetics and offer insights into the adaptive evolution of this rare species.

## Graphical abstract

We assembled and annotated the complete chloroplast genome of *L. yongfuensis*. The genome structure, gene content, and phylogenetic relationships were analyzed, providing new insights into the species' evolutionary history. These findings offer valuable genomic resources for future studies on taxonomy, conservation, and molecular breeding of *L. yongfuensis*.

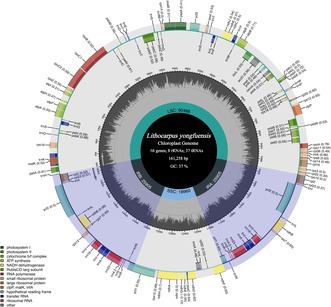

## 1 Introduction

The genus *Lithocarpus* (Fagaceae) is a species‐rich lineage, comprising approximately 340 species distributed across East Asian tropical and subtropical monsoon regions (POWO 2024; Strijk 2024). China is one of the key diversity and evolutionary centers for this genus, harboring 123 species, 69 of which are endemic (Huang et al. 1999). These trees, occurring across a broad elevational and latitudinal range, contribute significantly to regional forest ecosystem functions and ecological services (Huang et al. 1999).

*L. yongfuensis* Q. F. Zheng was first described in 1985 and is narrowly endemic to Zhangping, Fujian Province, southeastern China, where it inhabits mixed forests at approximately 850 m elevation (Zheng 1985; Huang et al. 1999). Fewer than 10 wild individuals are currently known (Liao et al. 2024), and the species is classified as critically endangered (CR) under the International Union for Conservation of Nature (IUCN) criteria (IUCN (Ed.) 2012). Despite its imperiled status, knowledge of *L. yongfuensis* remains limited to its type locality and basic morphological description, severely impeding conservation planning. Understanding a species' evolutionary background is fundamental to conservation biology. The chloroplast genome, owing to its uniparental inheritance, relatively conserved structure, and moderate mutation rate, has been widely used in plant systematics, phylogenetics, and population genetics (Balaji et al. 2025). Most angiosperm chloroplast genomes range from 120 to 160 kb, typically comprising a large single‐copy (LSC) region, a small single‐copy (SSC) region, and two inverted repeats (IRs) (Palmer 1982, 1985; Shinozaki et al. 1986). In addition to its value in phylogenetics, the chloroplast genome encodes genes essential for photosynthesis and other metabolic functions (Shinozaki et al. 1986; Wolfe et al. 1987; Yu et al. 2003). Patterns of chloroplast genome evolution can reveal adaptations to environmental stress, providing insights for species recovery strategies (Provan et al. 2001; Guo et al. 2020; Long et al. 2020; Chen et al. 2021; Tergemina et al. 2022). With the advent of high‐throughput sequencing, an increasing number of complete chloroplast genomes have become available for Fagaceae, including 34 species of *Lithocarpus* (Ma et al. 2021; Shelke et al. 2022; Wu et al. 2022; Fu et al. 2023; Yang et al. 2024). These resources provide a valuable comparative framework to investigate the maternal evolutionary history and chloroplast genome evolution of *L. yongfuensis*.

In this study, we sequenced, assembled, and annotated the complete chloroplast genome of *L. yongfuensis*. Our objectives were to: (i) characterize its structural and functional features, including gene content, repeat sequences, and codon usage; (ii) compare genome architecture and variation with related *Lithocarpus* species to detect divergence patterns; and (iii) reconstruct a plastome‐based phylogeny to clarify its evolutionary placement and assess signatures of adaptive evolution. These results provide a genomic foundation for future research on the conservation and evolutionary biology of this highly threatened species.

## 2 Materials and Methodology

### 2.1 Plant Sampling

On 22 November 2023, the field sampling of *L. yongfuensis* was conducted at Chang‐lun‐tou, Zhangping, Fujian Province, China (117.3641827° E, 25.1831042° N; altitude: 950 m). Two to three healthy leaves were collected from a single individual and immediately dried in silica gel for preservation until genomic DNA extraction. Permission for collecting the samples was not needed from the local relevant authority. The voucher specimen (L. F. Yang et al. *DM28035*) was deposited in the Herbarium of Xishuangbanna Tropical Botanical Garden, Chinese Academy of Sciences (HITBC). The acting deputy director of the specimen museum is Jianwu Li, and his email address is ljw@xtbg.org.cn.

### 2.2 Genome Extraction, Qualification, and Sequencing

We extracted high‐quality genomic DNA using a modified CTAB protocol (Doyle 1991). DNA concentration was quantified with a Qubit 4.0 Fluorometer (Invitrogen, Thermo Fisher Scientific, USA), and integrity was assessed by 1% (w/v) agarose gel electrophoresis. Paired‐end sequencing (PE150) was performed on the BGI T7 platform using the DNBSEQ‐T10 × 4RS sequencing kit (FCL PE150; Catalog No. 940‐000100‐00).

### 2.3 Chloroplast Genome Assembly and Annotation

Raw reads were filtered using Trimmomatic v0.36 with default parameters to remove adapter sequences and low‐quality bases (Bolger et al. 2014). The chloroplast genome was assembled de novo using GetOrganelle v1.7.5 (Jin et al. 2020) with the parameters “‐R 10‐k 21456585105115127‐F embplant_pt,” using *L. balansae* (GenBank accession: KP299291) as the reference. Genome annotation was performed with CpGAVAS and verified using DOGMA (http://dogma.ccbb.utexas.edu/) (Wyman et al. 2004; Liu et al. 2012), BLAST (Ye et al. 2006), and tRNAscan‐SE for tRNA gene identification (Chan and Lowe 2019). Manual corrections were made in Geneious Prime v2024.0.5 (Kearse et al. 2012), referring to *L. hancei* (NCBI accession: MW375417) and *L. balansae* (NCBI accession: KP299291) to confirm start/stop codons and exon–intron boundaries. The circular genome map was generated using OGDRAW v1.3.1 (Greiner et al. 2019).

### 2.4 Codon Usage Bias

Coding sequences (CDSs) were extracted using the RSCU command in CPStools v2.0.2 (Huang et al. 2024), filtering out sequences shorter than 300 bp or lacking an ATG start codon. Relative synonymous codon usage (RSCU) values were subsequently calculated (Huang et al. 2024). GC content at the first, second, and third codon positions (GC1, GC2, and GC3), as well as the overall GC content (GC_all), were computed using CodonW v1.4.4 (Peden 1999). Additionally, the effective number of codons (ENC), codon bias index (CBI), codon adaptation index (CAI), frequency of optimal codons (FOP), and codon counts were calculated with CodonW. RSCU values were visualized using the RSCU plotting tool on the online platform (http://cloud.genepioneer.com:9929/). Neutrality, ENC, and PR2‐bias plots were generated to evaluate codon usage patterns and their potential determinants (Wright 1990; Sueoka 1988, 1999).

### 2.5 Repeated Sequences

Simple sequence repeats (SSRs) were detected using the MISA online tool (https://webblast.ipk‐gatersleben.de/misa/) with thresholds set to 10, 5, 4, 3, 3, and 3 for mono‐, di‐, tri‐, tetra‐, penta‐, and hexanucleotide repeats, respectively (Beier et al. 2017). Tandem repeats were identified using Tandem Repeats Finder (TRF) v4.09 (https://tandem.bu.edu/trf/trf.html) with alignment parameters of match = 2, mismatch = 7, indels = 7, and a minimum alignment score of 80. Long terminal repeats, including forward, reverse, complement, and palindromic repeats, were detected using the REPuter online program (http://bibiserv.techfak.uni‐bielefeld.de/reputer/) with a minimum repeat size of 30 bp and a Hamming distance of 3 (Kurtz et al. 2001).

### 2.6 Comparative Genomic Analyses

We downloaded 34 complete chloroplast genomes of *Lithocarpus* from the National Center for Biotechnology Information (NCBI) and corrected annotation inconsistencies using Geneious Prime v9.0.2 (Kearse et al. 2012). Annotations were further validated with the gbccheck function in CPStools (Huang et al. 2024). Boundaries of the LSC, SSC, and IR regions were visualized using CPJSdraw (http://cloud.genepioneer.com:9929/#/tool/alltool/detail/335) (Li et al. 2023). Genome structural variation was assessed using Mauve within Geneious Prime (Kearse et al. 2012; Darling et al. 2010), and interspecific alignment was conducted with mVISTA, using *L. yongfuensis* as the reference (Frazer et al. 2004). Nucleotide diversity was analyzed using CPStools and visualized with ggplot2 in R (Wickham 2011).

### 2.7 Selection Pressure Analysis

To detect positive selection, the *K*a/*K*s (Hurst 2002) module in CPStools v2.0.2 (Huang et al. 2024) was used, with *L. yongfuensis* as the reference for comparison against 34 other *Lithocarpus* species. Protein‐coding genes (PCGs) with a *K*a/*K*s ratio greater than 1 were considered to be under positive selection. The results were visualized using the ggplot2 package in R (Wickham 2011).

### 2.8 Phylogenetic Reconstruction

A maximum likelihood (ML) phylogeny was reconstructed using 61 chloroplast genomes from Fagaceae, with *Betula cordifolia* (NCBI accession number MG386401) included as the outgroup to root the tree (Salojärvi et al. 2017; Zhou et al. 2022). Sequence alignment was performed using MAFFT v7.525 (Katoh and Standley 2013) and manually adjusted. The best‐fitting substitution model (K3Pu + F + R9) was selected by ModelFinder (Kalyaanamoorthy et al. 2017). The ML tree was constructed using IQ‐TREE, and branch support was assessed with 10,000 bootstrap replicates (Zhang et al. 2020). The resulting phylogenetic tree was visualized and edited in FigTree v1.4.4 (Rambaut 2018).

## 3 Results

**FIGURE 1:**
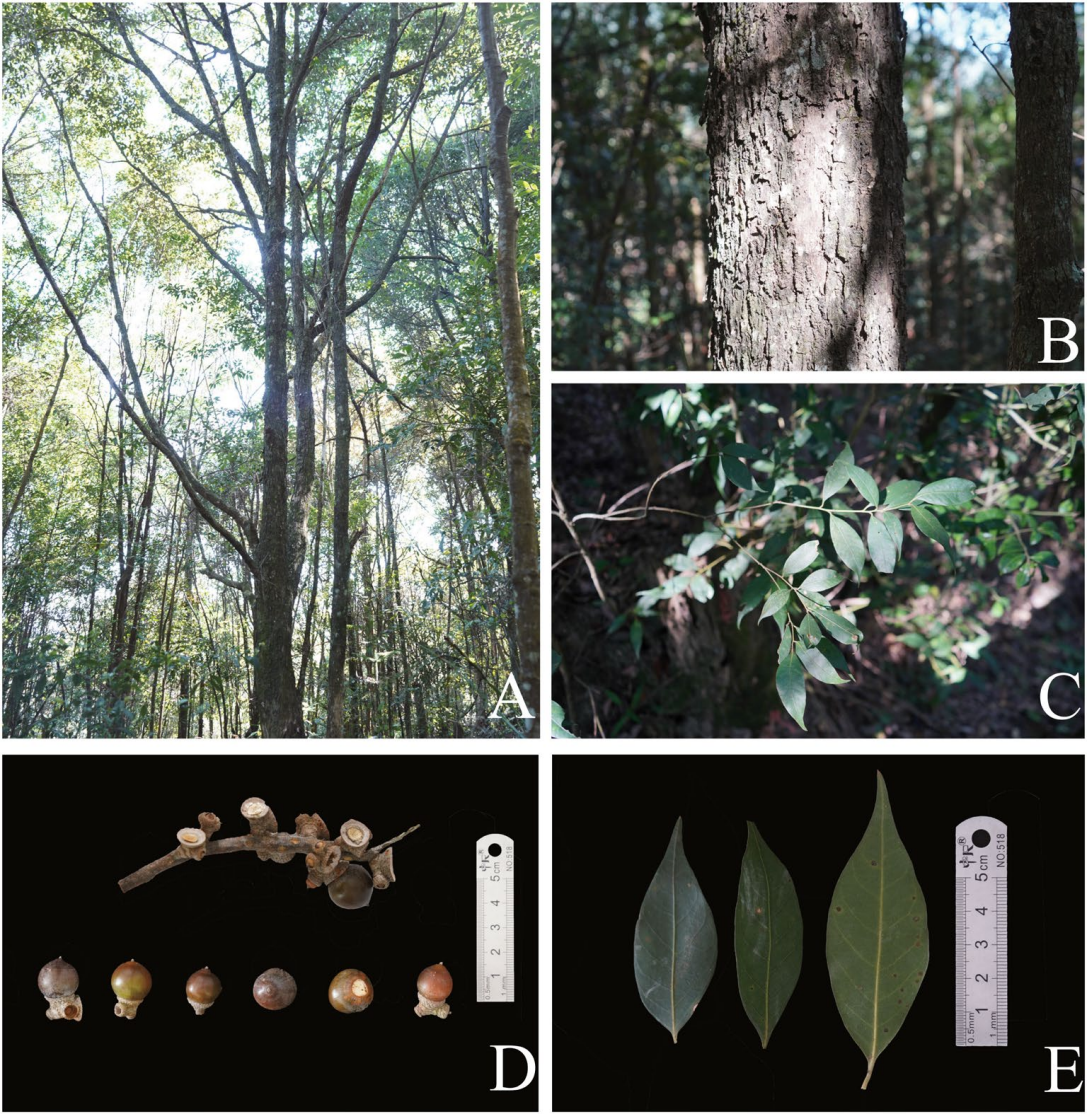
The photos of *L. yongfuensis*. (A) Photo of the entire plant. (B) Photo of *L*. *yongfuensis* trunk. (C) Photo of the leaves of *L*. *yongfuensis*. (D) Morphological characteristics of infructescence and fruits of *L*. *yongfuensis*. (E) Abaxial and adaxial sides of the mature leaves of *L*. *yongfuensis*.

The whole plant photo (Figure 1A), trunk image (Figure 1B), leaves image (Figure 1C), and the morphological characteristics of infructescence and fruits (Figure 1D) and the leaves (Figure 1E) of *L*. *yongfuensis* were shown in the figures.

### 3.1 Structural Characteristics of the Chloroplast Genome

**FIGURE 2:**
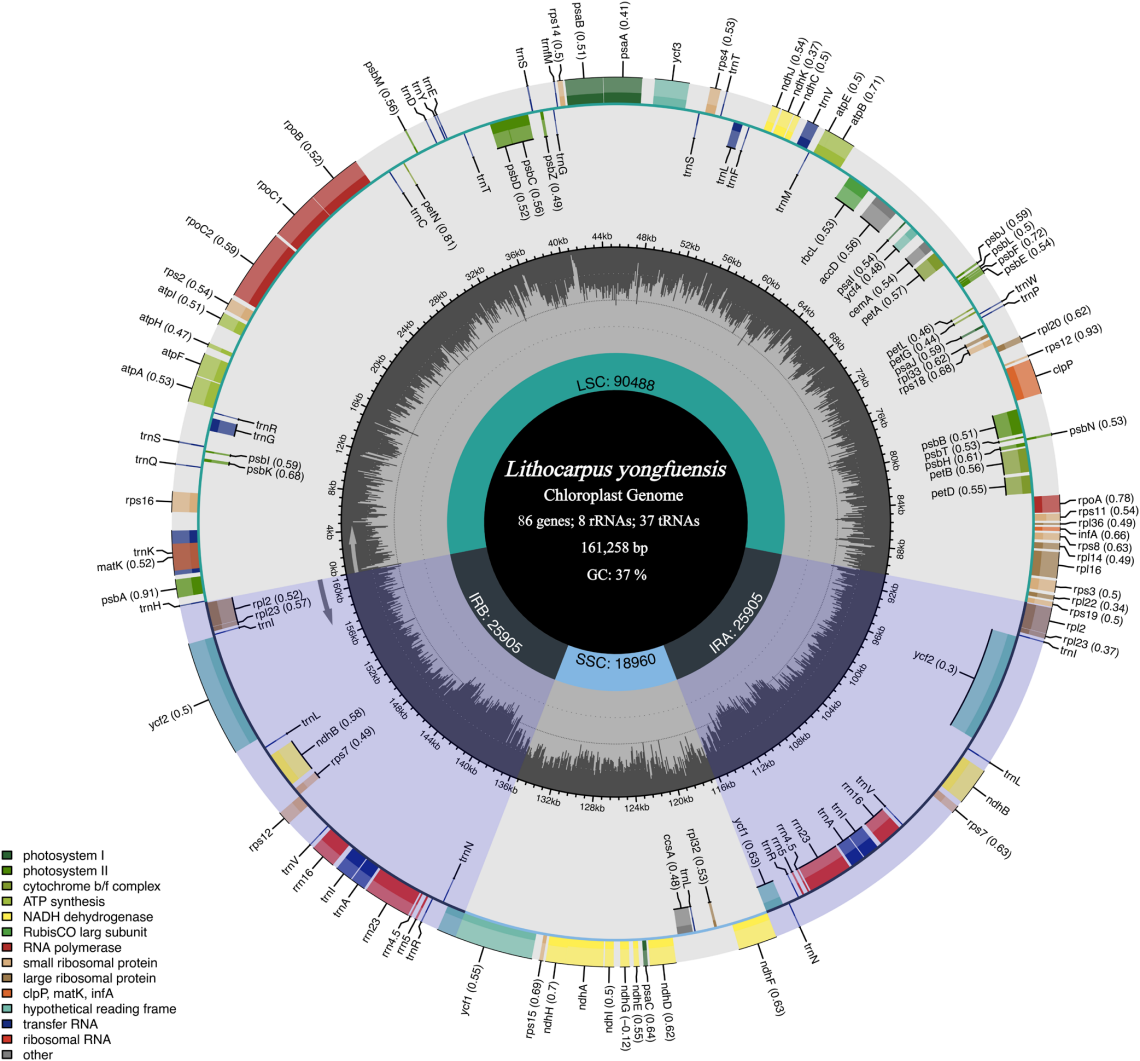
Chloroplast genome circular map of *L. yongfuensis*. The outermost circle shows the annotated genes, with those outside the circle transcribed clockwise and those inside transcribed counterclockwise. Genes are color‐coded by functional groups. The inner circle indicates the lengths and boundaries of the LSC, SSC, and two IR regions. The dark gray area within the inner circle represents the GC content. IR, inverted repeat; LSC, large single copy; SSC, small single copy.

The chloroplast genome of *L. yongfuensis* exhibits a typical quadripartite structure, comprising a LSC region, a SSC region, and two IRs. The complete chloroplast genome is 161,258 bp in length, including a 90,488 bp LSC, an 18,960 bp SSC, and a pair of IRs, each measuring 25,905 bp (Figure 2). The overall GC content is 36.8%, with corresponding values of 34.8%, 30.8%, and 42.7% for the LSC, SSC, and IR regions, respectively (Table S1).

A total of 131 genes were annotated, including 86 PCGs, 37 transfer RNA (tRNA) genes, and 8 ribosomal RNA (rRNA) genes. Within the IR regions, eighteen genes were duplicated, comprising 7 PCGs, 7 tRNAs, and 4 rRNAs. Among these, nine PCGs (*rps16*, r*pl2*, *rpl16*, *rpoC1*, *atpF*, *ndhA*, *ndhB*, *petB*, and *petD*) and six tRNAs (*trnA‐UGC*, *trnI‐GAU*, *trnV‐UAC*, *trnL‐UAA*, *trnG‐GCC*, and *trnK‐UUU*) each contained a single intron, while three PCGs (*ycf3*, *clpP*, and *rps12*) contained two introns (Table 1).

**TABLE 1:** Genetic classification of the chloroplast genome of *L. yongfuensis*.

Category	Gene group	Gene name
Photosynthesis	Subunits of photosystem I	*psaA*, *psaB*, *psaC*, *psaI*, *psaJ*
	Subunits of photosystem II	*psbA*, *psbB*, *psbC*, *psbD*, *psbE*, *psbF*, *psbH*, *psbI*, *psbJ*, *psbK*, *psbL*, *psbM*, *psbN*, *psbT*, *psbZ*
	Subunits of NADH dehydrogenase	ndhA*, ndhB* (2), *ndhC*, *ndhD*, *ndhE*, *ndhF*, *ndhG*, *ndhH*, *ndhI*, *ndhJ*, *ndhK*
	Subunits of cytochrome b/f complex	*petA*, petB*, petD*, *petG*, *petL*, *petN*
	Subunits of ATP synthase	*atpA*, *atpB*, *atpE*, atpF*, *atpH*, *atpI*
	Large subunit of rubisco	*rbcL*
	Subunits photochlorophyllide reductase	—
Self‐replication	Proteins of large ribosomal subunit	*rpl14*, rpl16*, rpl2* (2), *rpl20*, *rpl22*, *rpl23* (2), *rpl32*, *rpl33*, *rpl36*
	Proteins of small ribosomal subunit	*rps11*, rps12** (2), *rps14*, *rps15*, rps16*, *rps18*, *rps19*, *rps2*, *rps3*, *rps4*, *rps7*(2), *rps8*
	Subunits of RNA polymerase	*rpoA*, *rpoB*, rpoC1*, *rpoC2*
	Ribosomal RNAs	*rrn16S* (2), *rrn23S* (2), *rrn4.5*(2), *rr*n5 (2)
	Transfer RNAs	trnA‐UGC*(2), *trnC‐GCA*, *trnD‐GUC*, *trnE‐UUC*, *trnF‐GAA*, trnG‐GCC*, *trnG‐UCC*, *trnH‐GUG*, *trnI‐CAU* (2), trnI‐GAU* (2), trnK‐UUU*, *trnL‐CAA* (2), trnL‐UAA*, *trnL‐UAG*, *trnM‐CAU*, *trnN‐GUU* (2), *trnP‐UGG*, *trnQ‐UUG*, *trnR‐ACG* (2), *trnR‐UCU*0, *trnS‐GCU*, *trnS‐GGA*, *trnS‐UGA*, *trnT‐GGU*, *trnT‐UGU*, *trnV‐GAC* (2), trnV‐UAC*, *trnW‐CCA*, *trnY‐GUA*, *trnfM‐CAU*
Other genes	Maturase	*matK*
	Protease	clpP**
	Envelope membrane protein	*cemA*
	Acetyl‐CoA carboxylase	*accD*
	c‐type cytochrome synthesis gene	*ccsA*
	Translation initiation factor	*infA*
	other	—
Genes of unknown function	Conserved hypothetical chloroplast ORF	*ycf1* (2), *ycf2* (2), ycf3**, *ycf4*

*Note:* Gene*: Gene with one intron; Gene**: Gene with two introns; Gene (2): Number of copies of multi‐copy genes.

### 3.2 Repeated Sequences of the Chloroplast Genome

**FIGURE 3:**
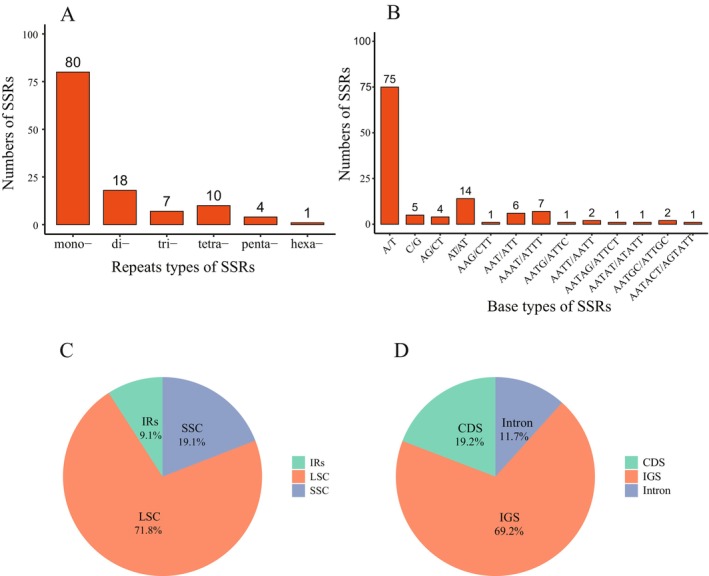
Types, distributions, and numbers of simple sequence repeats (SSRs) in the chloroplast genome of *L. yongfuensis*. (A) Number of SSRs categorized by six repeat types: Mono‐ (mononucleotides), di‐ (dinucleotides), tri‐ (trinucleotides), tetra‐ (tetranucleotides), penta‐ (pentanucleotides), and hexa‐ (hexanucleotides). (B) Number of SSRs by base composition. (C, D) Distribution of SSRs across different regions of the chloroplast genome. CDS, coding sequence; IGS, intergenic spacer; IRs, inverted repeats; LSC, large single‐copy; SSC, small single‐copy.

A total of 120 simple sequence repeats (SSRs) were identified in the chloroplast genome of *L. yongfuensis* (Figure 3A), comprising 13 distinct motif types (Figure 3B). Mononucleotide repeats were the most abundant, while hexanucleotide repeats were the least common, accounting for approximately 1% of all detected SSRs (Figure 3A). The SSRs exhibited a strong A/T base bias, with A/T‐rich motifs significantly outnumbering G/C‐rich motifs (Figure 3B). Most SSRs were located in the LSC and intergenic spacer (IGS) regions, whereas relatively few were found within the IR and coding regions (Figure 3C,D).

**FIGURE 4:**
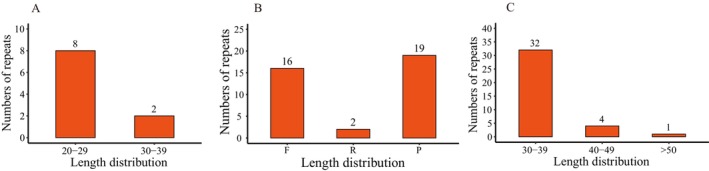
Distribution of the number and length of tandem and dispersed repeats in the chloroplast genome of *L. yongfuensis*. (A) Length distribution of tandem repeats; (B) Number of dispersed repeats; (C) Length distribution of dispersed repeats. F, forward repeats; P, palindromic repeats; R, reverse repeats.

In the chloroplast genome of *L. yongfuensis*, 10 tandem repeats were detected, with most repeat units ranging from 20 to 29 base pairs in length (Figure 3A). Among the four types of dispersed repeats, palindromic (19) and forward repeats (16) were the most abundant, whereas only two IRs were identified, and no complementary repeats were detected (Figure 4B). The lengths of dispersed repeat sequences ranged from 30 to 62 bp, with the majority concentrated in the 30–39 bp range (Figure 4C).

### 3.3 Codon Usage Bias of the Chloroplast Genome

**FIGURE 5:**
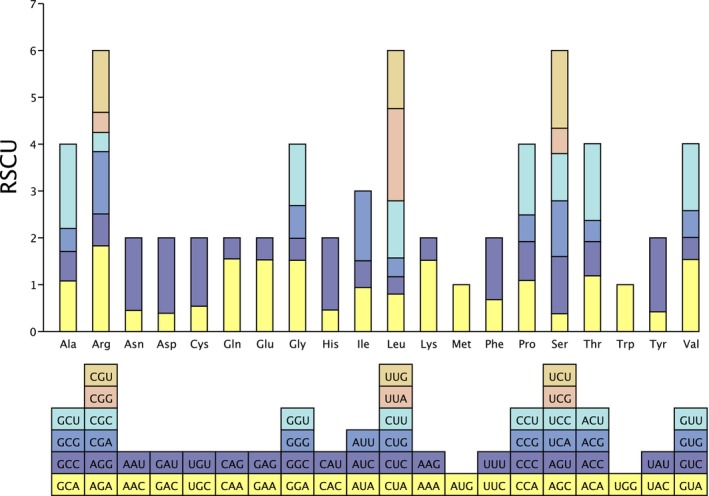
RSCU analysis of the chloroplast genome of *L. yongfuensis*.

CodonW analysis revealed that the PCGs in the chloroplast genome of *L. yongfuensis* encoded a total of 20,781 codons (Table S1). The GC content and codon bias indices indicated a relatively weak codon usage bias in this genome (Table S3). RSCU analysis showed that 30 out of 59 synonymous codons had RSCU values greater than 1, indicating preferential usage. Among these, only two codons (UUG and UCC) ended with G or C, while the remaining 28 codons ended with A or U (Table S3). Leucine (Leu), arginine (Arg), and serine (Ser) exhibited the highest levels of codon usage bias among all encoded amino acids (Figure 5).

**FIGURE 6:**
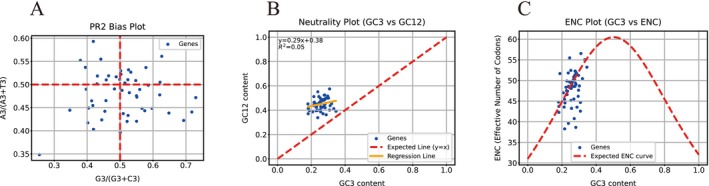
PR2‐bias, neutrality, and ENC plot analyses of *L*. *yongfuensis*. (A) PR2‐bias plot analysis; (B) Neutrality plot analysis; (C) ENC plot analysis of the chloroplast genome of *L. yongfuensis*.

To further investigate the factors influencing codon usage, PR2‐bias, neutrality, and ENC plot analyses were performed. The PR2‐bias plot revealed an uneven distribution of the four bases at the third codon position (A3 ≠ T3, G3 ≠ C3) across the four regions, indicating that codon usage is affected by nucleotide composition asymmetry (Figure 6A). Neutrality plot analysis showed a weak positive correlation between GC12 and GC3, although the correlation was not statistically significant (Figure 6B). The ENC plot (Figure 6C) indicated that while some genes clustered near the expected curve, others fell below it, suggesting that both mutation pressure and natural selection contribute to shaping codon usage bias in *L. yongfuensis*.

### 3.4 Comparative Chloroplast Genomic Analyses

The boundaries of the LSC/Irb, SSC/Ira, and Ira/LSC junctions were generally conserved across the sampled *Lithocarpus* species. Specifically, the LSC/Irb boundary consistently involved *rps19* and *rpl2*; the SSC/Ira boundary occurred within the *ycf*1 gene; and the Ira/LSC boundary was defined by *rpl2* and *trnH*. In *L. yongfuensis*, the *rps19* gene shifted 10 bp from the LSC/Irb junction (JLB), and the *ycf1* gene at the SSC/Ira boundary (JSA) contracted by 4581 bp—a pattern similar to that observed in *L. crassifolius* and *L. brevicaudatus* (Figure S1).

The chloroplast genomes of *Lithocarpus* exhibited strong collinearity in the overall structure and gene order, with no evidence of gene rearrangements or inversions (Figure S2). Nevertheless, several regions showed higher sequence divergence. mVISTA analysis revealed one highly variable exon (*ndhA*) and four IGS regions (*psbA*‐*trnH*, *rps16‐trnK‐UUU*, *accD‐psaI*, and *rpl32‐ndhF*) exhibiting greater sequence variation than the rest of the genome. As expected, non‐coding and single‐copy (SC) regions displayed higher nucleotide diversity compared to coding and IR regions (Figure S3).

**FIGURE 7:**
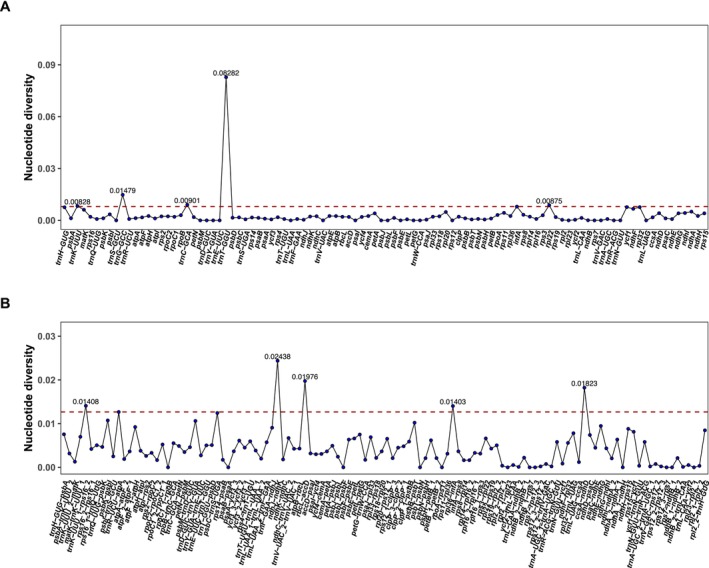
Nucleotide polymorphism analysis of 35 *Lithocarpus* chloroplast genomes. The X‐axis represents nucleotide positions, and the Y‐axis shows Pi values. Genes or IGS with high Pi values were labeled with specific numerical values. (A) Nucleotide polymorphism in CDS regions. (B) Nucleotide polymorphism in IGS regions.

To quantify sequence variability, nucleotide diversity (Pi) values were calculated across the chloroplast genomes. Pi values ranged from 0 to 0.08282, with twelve highly variable regions (e.g., *trnK‐UUU_1‐rps16*_2, *psbK‐psbI*, *trnR‐UCU‐atpA*, *trnD‐GUC‐trnY‐GUA*, *psbC‐trnS‐UGA*, *ndhJ‐ndhK*, *rbcL‐accD*, *psbB‐psbT*, *rpl36‐infA*, *ccsA‐ndhD*, *trnG‐GCC*, and *trnT‐GGU*) showing Pi values greater than 0.01 (Table S4). The most variable region was the *trnT‐GGU* region, with the highest Pi value of 0.08282 (Figure 7).

### 3.5 Phylogenetic Analysis

**FIGURE 8:**
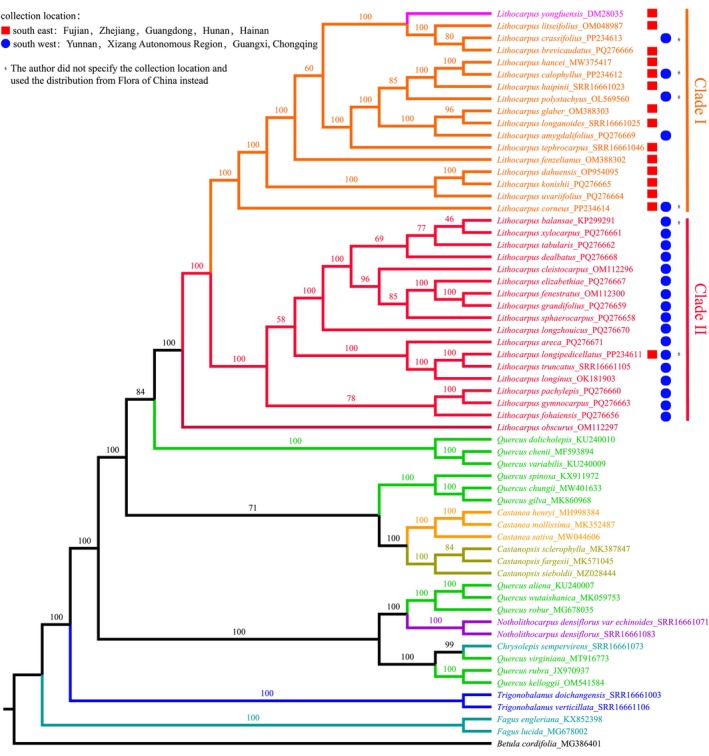
ML phylogenetic tree of Fagaceae based on chloroplast genomes. *Betula cordifolia* (NCBI accession MG386401) was used as the outgroup to root the tree. Numbers above the branches indicate bootstrap support values. Each species of the genus *Lituocarpus* was marked with red or blue circles indicating the collection regions. The two major clades of species derived from the genus *Lithocarpus* were designated as Clade I and Clade II.

The phylogenetic tree reconstructed from whole chloroplast genome sequences of 61 taxa was well resolved, with the main tree topology supported by high bootstrap values ranging from 46 to 100 (Table S2). The monophyly of most Fagaceae genera was strongly supported, except for *Quercus*, which exhibited a polyphyletic pattern. Within *Lithocarpus*, *L. obscurus* was identified as the earliest diverging lineage. The remaining species formed two major clades (Clade I and Clade II) (Figure 8). *L. yongfuensis* clustered within Clade II, forming a sister group relationship with [*L. litseifolius* + ( *L. crassifolius* + *L. brevicaudatus*)], indicating a close evolutionary relationship among these species (Figure 8).

### 3.6 Selective Pressure Analysis

**FIGURE 9:**
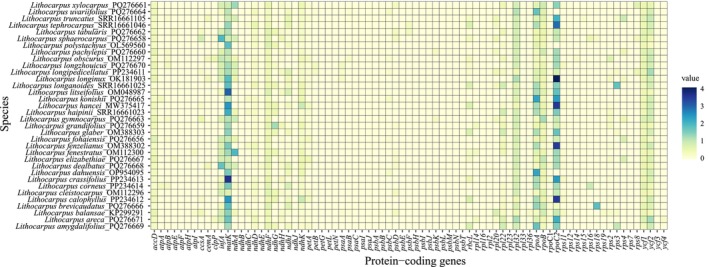
Heatmap of pairwise *K*a/*K*s ratios for chloroplast PCGs between *L. yongfuensis* and other *Lithocarpus* species. The color scale legend on the right indicates the corresponding *K*a/*K*s ratio values.

Most PCGs in the chloroplast genome of *L. yongfuensis* exhibited *K*a/*K*s ratios below 1, with many showing ratios of 0, indicating that purifying selection predominates across the chloroplast genome (Figure 9). Notably, the *K*a/*K*s ratios of the *rpoC2* and *matK* genes exceeded 1 in several species, suggesting that these genes may have undergone positive selection during evolution.

## 4 Discussion

### 4.1 Chloroplast Genome Characteristics of *L. yongfuensis*

With the accumulation of molecular resources for endangered species (Avise 1989), chloroplast genomes have become important tools for elucidating the systematic relationships and conservation genetics (Balaji et al. 2025). The chloroplast genome structure of Fagaceae is highly conserved, with no significant rearrangements or large‐scale deletions observed in several genera such as *Quercus*, *Castanopsis*, and *Lithocarpus* (Shi et al. 2025; Yang et al. 2024). Our results showed that core parameters such as genome size, GC content, the proportions of LSC, SSC, and IR regions, and gene order were largely consistent, with no obvious structural variations. This indicates that Fagaceae chloroplast genomes have been subject to strong purifying selection during evolution, which has maintained their structural and functional stability (Zhou et al. 2024). Such structural conservation facilitates the identification of orthologous genes both within and between genera (Fulton et al. 2002) and provides reliable data for phylogenetic analysis and species identification.

Additionally, the cpSSR features of *L. yongfuensis* were also highly conserved. The number of SSRs, the predominant repeat motifs (mainly mononucleotides), and their genomic distribution closely matched those of other *Lithocarpus* species, exhibiting significant collinearity. Similar conservation has been reported in other Fagaceae genera (Zhou et al. 2024), and cpSSRs have been shown to be broadly applicable in intergeneric phylogenetics and population genetic structure analyses (Provan et al. 2001; Pezoa et al. 2021). Although the low variability of SSRs may limit their resolution at the intraspecific level (Šarhanová et al. 2018), screening for highly polymorphic loci across genera holds promise for developing universal molecular markers in Fagaceae, thereby improving species identification and germplasm conservation efficiency (Chen et al. 2023).

Despite the overall structural conservation, certain photosynthesis‐related genes of *L. yongfuensis* exhibited localized variation. Preliminary analyses indicated that the *rpoC2* and *matK* genes may be under positive selection, suggesting potential functional adaptation to the specific ecological conditions (Levasseur et al. 2007). The *rpoC2* gene encodes a subunit of RNA polymerase that may regulate the expression of genes such as *psbA*, influencing photosystem II activity and thus adaptation to shaded understory environments (Jin et al. 2022, 2024; Wu et al. 2023). Meanwhile, *matK* is broadly involved in the splicing of group II introns in chloroplasts, crucial for the correct expression of photosynthetic and metabolic genes like *rpl2* (Zoschke et al. 2010). Considering that *L. yongfuensis* inhabits the shaded understory of southern evergreen broadleaf forests characterized by low light, high humidity, and microclimatic fluctuations (Zheng 1985; Huang et al. 1999), these gene‐level fine‐tunings may reflect adaptive responses, highlighting the genetic distinctiveness of this germplasm. Furthermore, nucleotide polymorphism and mVISTA analyses jointly revealed high variability in the *trnK‐rps16* intergenic spacer among *Lithocarpus* species, supporting its potential as a candidate DNA barcode. This region has been demonstrated to possess good phylogenetic resolution in several Fagaceae genera, especially for groups with blurred boundaries or frequent hybridization (Yang et al. 2016; Ye et al. 2019; Zhou et al. 2021). Therefore, integrating highly polymorphic SSRs with specific intergenic spacers will facilitate more efficient species delimitation for *L. yongfuensis* and its close relatives, supporting subsequent endangered species monitoring and germplasm management.

### 4.2 Phylogeny of Fagaceae

Based on the molecular phylogenetic tree constructed from complete cpDNA, our results support the monophyly of the genus *Lithocarpus*. Consistent with previous studies, we found that *Quercus* is polyphyletic, likely as a result of extensive ancient hybridization and introgression events during its evolutionary history (Yang et al. 2024). The two main *Lithocarpus* clades (Clade I and Clade II) recovered in this study correspond to geographic differentiation between southwest and southeast China, respectively, reflecting a clear phylogeographic pattern consistent with prior reports (Yang et al. 2024). Such geographic structure likely reflects restricted seed dispersal within the complex topography of these regions.

Notably, *L. yongfuensis* clustered with three other *Lithocarpus* species (*L. litseifolius*, *L. crassifolius* , and *L. brevicaudatus* ) into a small clade. However, *L. crassifolius* , which is distributed in southwestern China, is geographically distant from the other three species (Huang et al. 1999). Although these species share several morphological traits (e.g., coriaceous leaves, pubescent leaf surfaces, lanceolate leaf shapes, and acorns with depressed scars) (Chen et al. 2023), frequent hybridization, introgression, and incomplete lineage sorting events within *Lithocarpus* hinder chloroplast phylogenies from fully reflecting species evolutionary relationships (Xu et al. 2024; Huang et al. 1999). Therefore, the evolutionary relationships of *L. yongfuensis* and its close relatives require further clarification using high‐throughput nuclear genomic sequencing.

### 4.3 Conservation Implications for *L. yongfuensis*

*L. yongfuensis* is known globally only from a fragmented mixed forest remnant at approximately 850 m elevation in Zhangping, Fujian Province, with an extremely small population size (fewer than 10 wild individuals) and high habitat fragmentation (Zheng 1985; Huang et al. 1999). Given the elevated variation and evidence of positive selection in certain chloroplast photosynthesis‐related genes, this germplasm exhibits distinct genetic characteristics warranting further conservation efforts.

The southeastern coastal region of China is the distribution center for *Lithocarpus* species, with Fagaceae being dominant forest components and multiple *Lithocarpus* species often co‐occurring (POWO 2024). Hybridization and introgression between rare *Lithocarpus* species and more widespread congeners in this region may lead to genetic swamping and extinction of rare species (Levin et al. 1996). In habitats with low human disturbance, spatial crowding often effectively prevents or reduces interspecific hybridization, thereby maintaining the genetic distinctiveness of rare species (De Lange and Norton 2004). Thus, in situ conservation of *L. yongfuensis* habitats is critically important. Moreover, the acorns of *Lithocarpus* species have thick pericarps and are frequently consumed by animals (Xiao and Zhang 2006; Zhang et al. 2006; Chen et al. 2020), resulting in low natural seedling recruitment. Therefore, ex situ conservation efforts, including seedling cultivation and subsequent reintroduction, are also vital strategies to maintain and restore *L. yongfuensis* populations.

## 5 Conclusions

This study has successfully elucidated, for the first time, the complete chloroplast genome of *L. yongfuensis* and compared it with those of 34 other *Lithocarpus* species from NCBI, providing key data for clarifying its taxonomic placement and exploring the mechanisms underlying its endangered status. The results showed that the chloroplast genome of *L. yongfuensis* closely resembled those of other *Lithocarpus* species in quadripartite structure, GC content, codon usage patterns, and gene order. The variable locus *rps16–trnK‐UUU* in *L. yongfuensis* had potential for developing species‐specific molecular identification primers. Phylogenetic analysis based on chloroplast genomes placed *L. yongfuensis* within the southeastern China clade, closely related to *L. crassifolius* , *L. litseifolius*, and *L. brevicaudatus* . Additionally, the genes *matK* and *rpoC2* were identified as being under positive selection. *matK* functions in RNA splicing, and *rpoC2* contributes to chloroplast transcription regulation. These findings provide a preliminary understanding of its phylogenetically close relatives and offer critical foundational data for the conservation genetics and adaptive evolution of this species.

## Author Contributions

**Fengzhi Gu:** data curation (equal), formal analysis (equal), methodology (lead), resources (lead), software (equal), validation (lead), visualization (equal), writing – original draft (lead). **Lifang Yang:** conceptualization (lead), data curation (equal), formal analysis (equal), funding acquisition (lead), software (equal), visualization (equal), writing – review and editing (lead).

## Funding

This work was supported by the National Natural Science Foundation of China (32460060 and 31972858). Yunnan Key Laboratory for Integrative Conservation of Plant Species with Extremely Small Populations (PSESP2021F01).

## Conflicts of Interest

The authors declare no conflicts of interest.

## Supporting information


**Figure S1:** Comparison of the junction regions (JLA, JLB, JSB, and JSA) among 35 cp genomes of *Lithocarpus* section.
**Figure S2:** Mauve alignment of 35 cp genomes of *Lithocarpus* section.
**Figure S3:** The comparison of *Lithocarpus* chloroplasts with the mVISTA program.
**Table S1:** Codon parameter characterization.
**Table S2:** Genebank data of plants used in comparative genomic analyses and the phylogenetic tree of this study.
**Table S3:** The RSCU values in cp genomes of *L*. *yongfuensis*.
**Table S4:** Nucleotide polymorphism analysis of 35 *Lithocarpus* chloroplast genomes.

## Data Availability

The data that support the findings of this study are openly available in NCBI and will be released after acceptance (https://www.ncbi.nlm.nih.gov/). The complete chloroplast genome of *L. yongfuensis* was deposited in GenBank under the accession PX109668 (https://www.ncbi.nlm.nih.gov/nuccore/PX109668). The biological sample number is DM28035. All additional data are provided within the Supporting Information.
