# Supplementary material for: Structural Characteristics, Comparative Analyses, and Conservation Significance of the Complete Chloroplast Genome of the Critically Endangered Lithocarpus yongfuensis (Fagaceae)
Source: Ecol Evol. 2026 Jan 9;16(1):e72833. doi: 10.1002/ece3.72833 (PMC12789196; doi:10.1002/ece3.72833)
Supplement: Supplementary file 1 — Figure S1: Comparison of the junction regions (JLA, JLB, JSB, and JSA) among 35 cp genomes of Lithocarpus section. Figure S2: Mauve alignment of 35 cp genomes of Lithocarpus section. Figure S3: The comparison of Lithocarpus chloroplasts with the mVISTA program. Table S1: Codon parameter characterization. Table S2: Genebank data of plants used in comparative genomic analyses and the phylogenetic tree of this study. Table S3: The RSCU values in cp genomes of L. yongfuensis. Table S4: Nucleotide polymorphism analysis of 35 Lithocarpus chloroplast genomes. [file ECE3-16-e72833-s001.docx]

**Figure S1.** Comparison of the junction regions (JLA, JLB, JSB, JSA) among 35 cp genomes of *Lithocarpus* section.


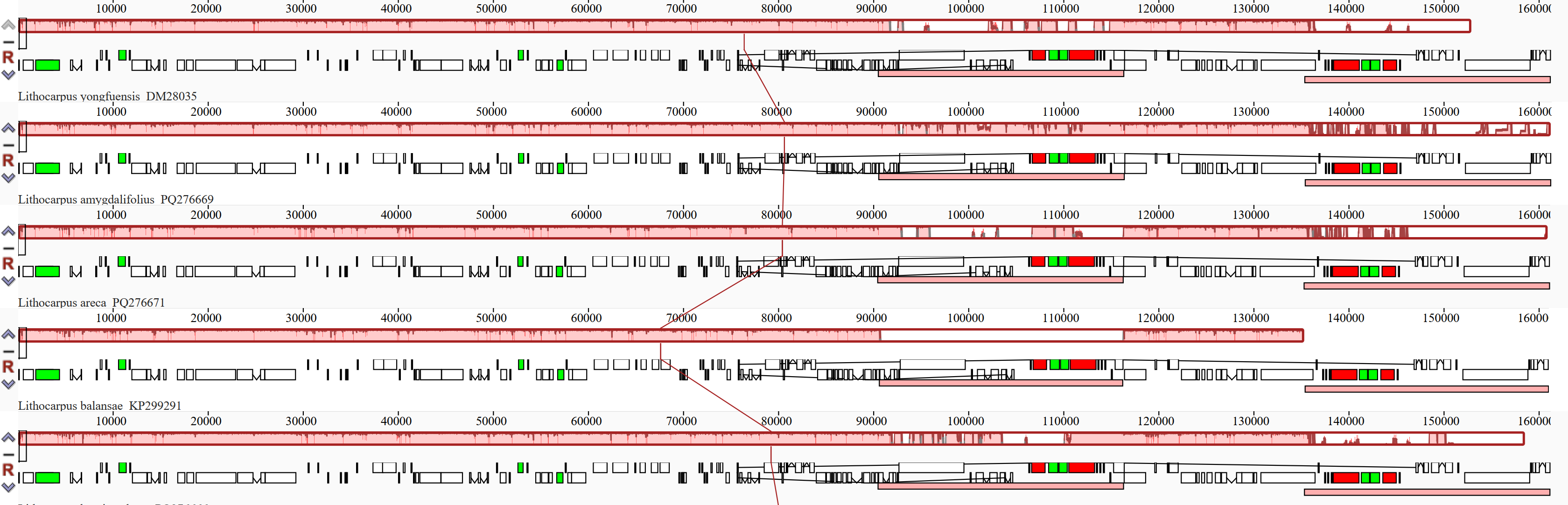


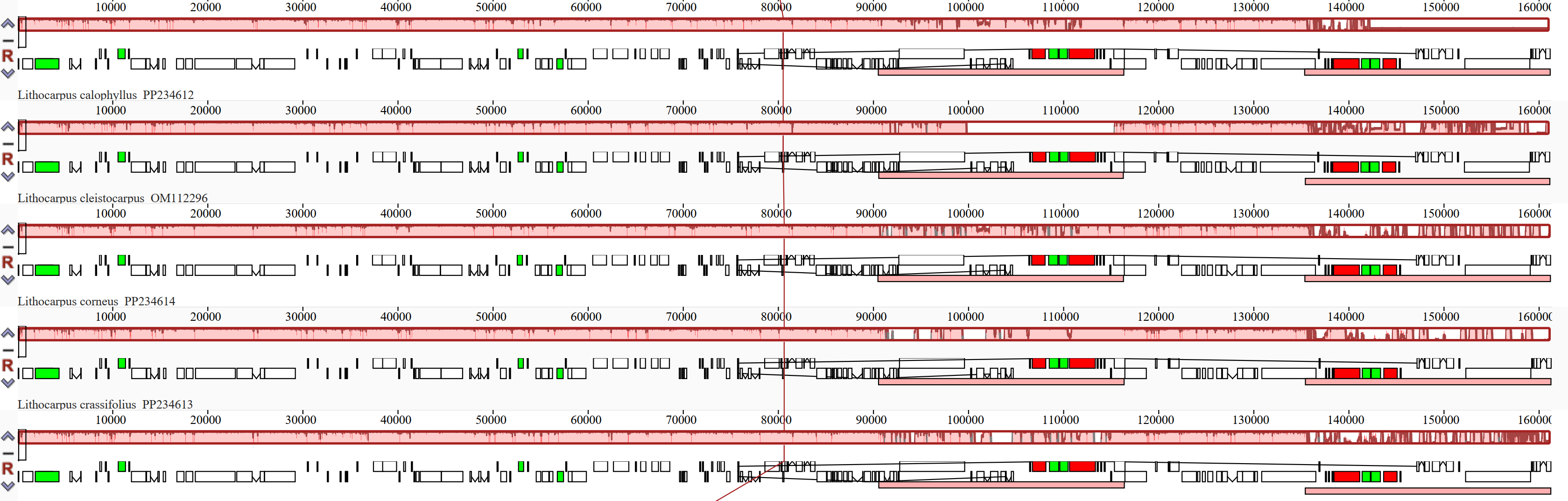


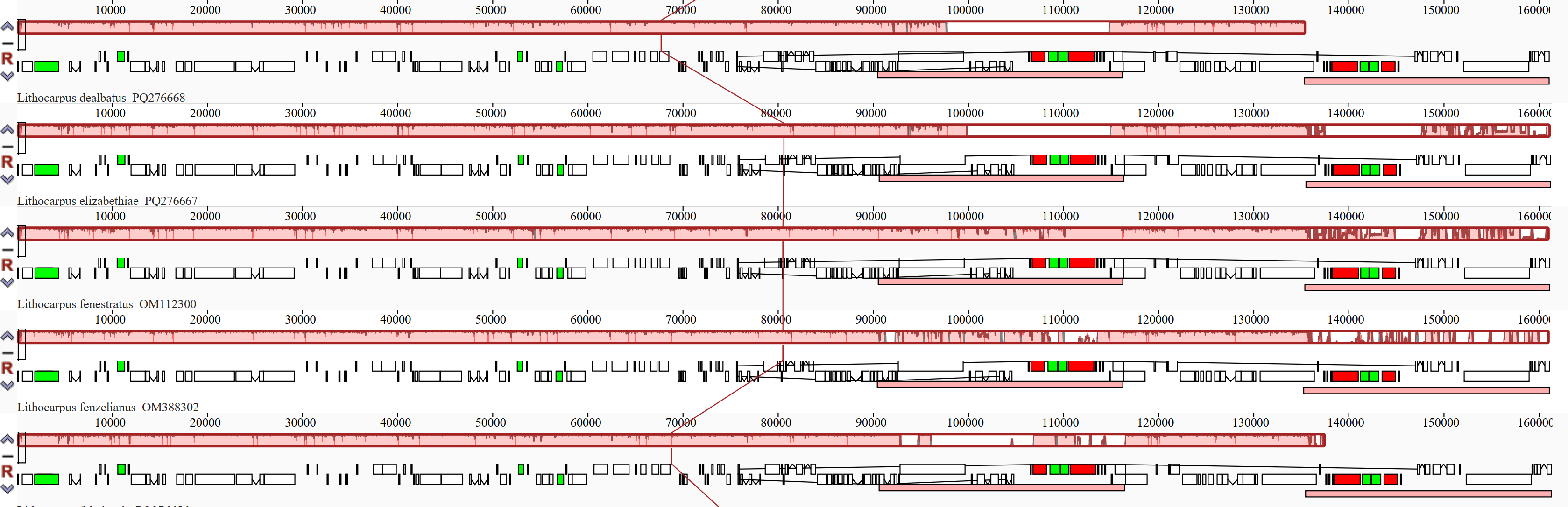


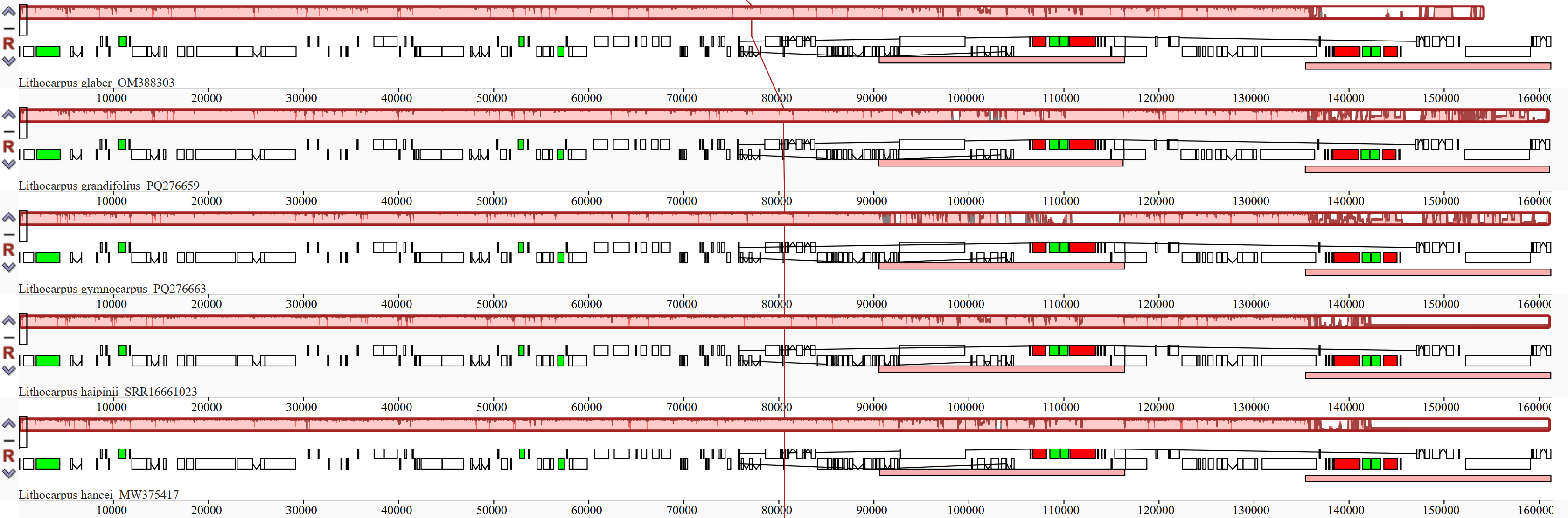


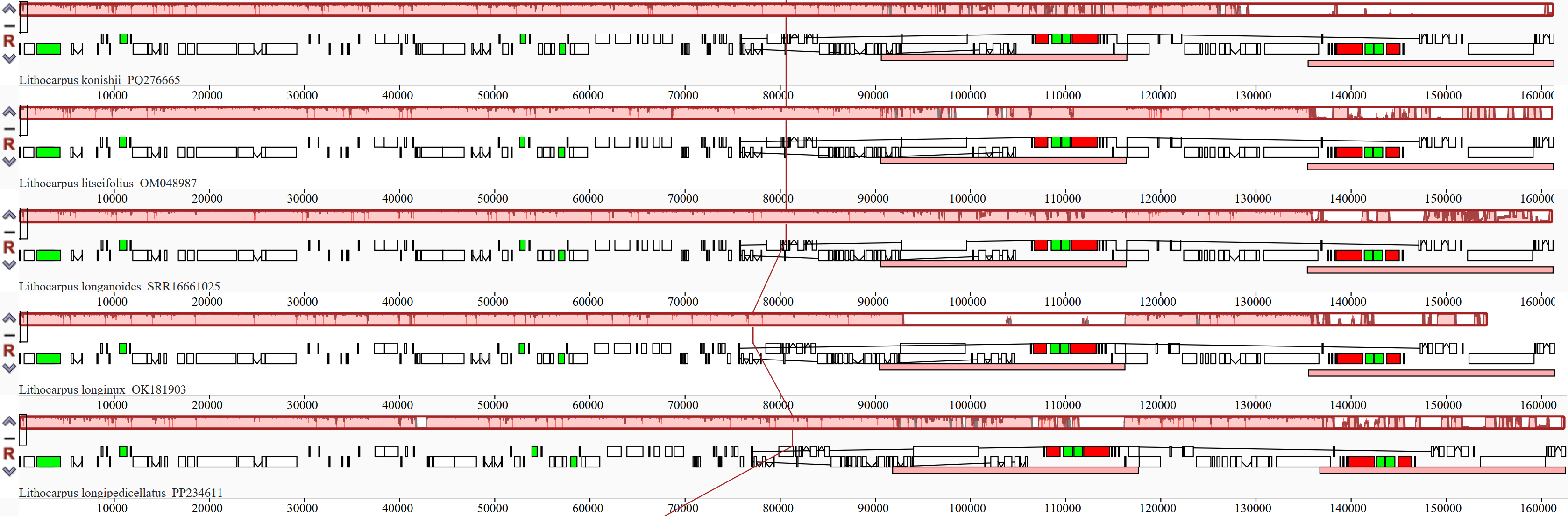


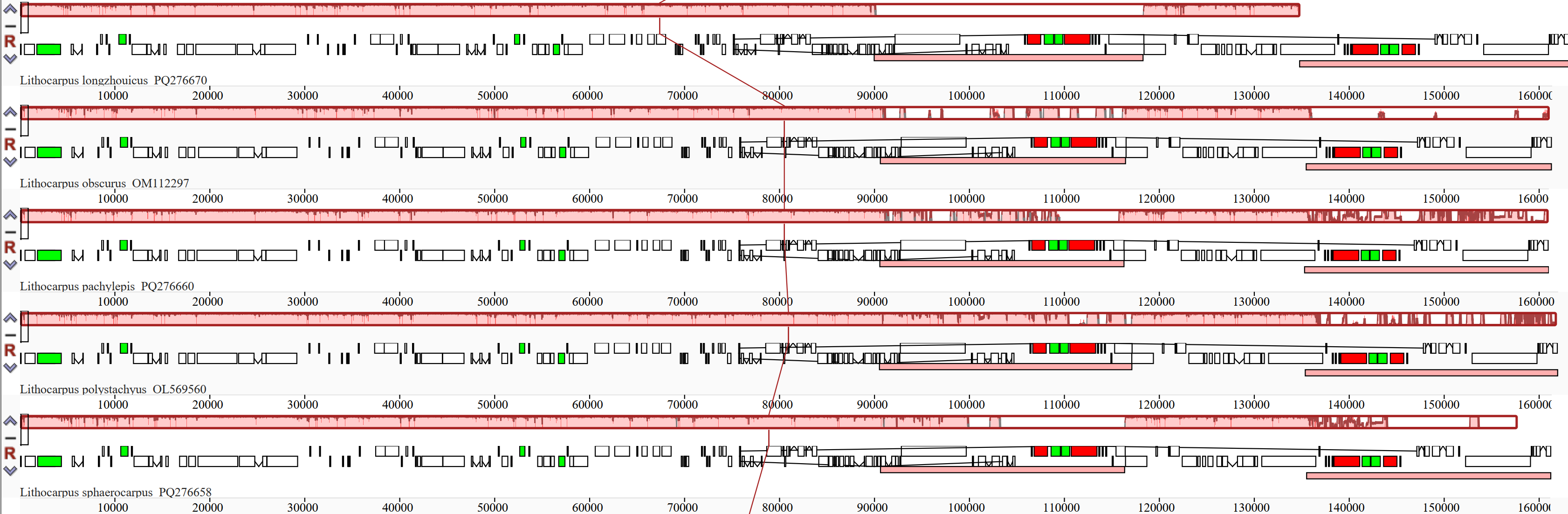


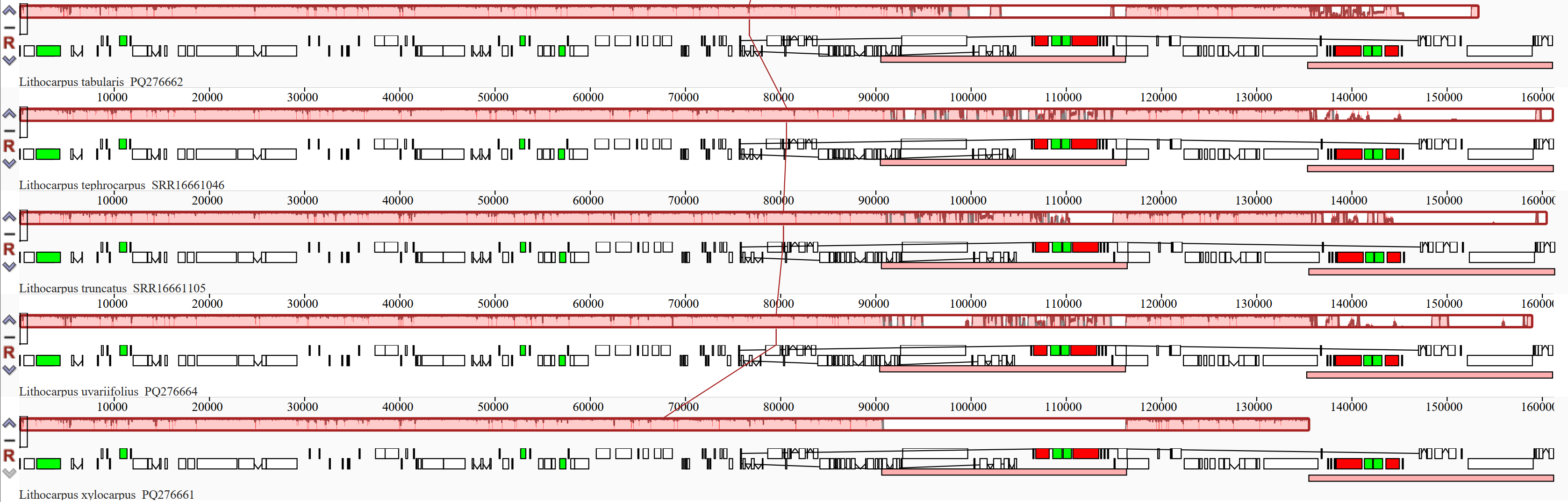


**Figure S2.** Mauve alignment of 35 cp genomes of *Lithocarpus* section*.* The box structure below the genome represents the corresponding gene annotation information: the white rectangle represents PCGs, the red rectangle represents rRNAs, and the green rectangle represents tRNAs. The introns are connected by line segments.





Figure S3. The comparison of *Lithocarpus plastomes* with the mVISTA program, with *L. corneus* designated as the reference. The percentage of identity is shown in the vertical axis, ranging from 50 to 100%, while the horizontal axis shows the position within the plastome. Genome regions are color-coded as coding, tRNA and rRNA, non-coding, and mRNA sequences

**Table S1.** Codon parameter characterization of the cp genomes of *L. yongfuensis.*

| **Species** | **GC1（%）** | **GC2（%）** | **GC3**  **(%)** | **GC_all(%)** | **GC3s(%)** | **ENC** | **CAI** | **CBI** | **FOP** | **No.of codons** |
| --- | --- | --- | --- | --- | --- | --- | --- | --- | --- | --- |
| *Lithocarpus yongfuen* | 50.51 | 36.04 | 29.44 | 37.97 | 25 | 48.94 | 0.1698 | 0.0094 | 0.3544 | 20781 |

**Table S2.** Genebank data of plants used in comparative genomic analyses and the phylogenetic tree of this study.

| **Taxa** | **Family** | **Genus** | **GenBank Accession** |
| --- | --- | --- | --- |
| *Lithocarpus yongfuensis* Q. F. Zheng | Fagaceae | *Lithocarpus* | PX109668 |
| *L. litseifolius* (Hance) Chun | Fagaceae | *Lithocarpus* | OM048987 |
| *L. crassifolius* A. Camus | Fagaceae | *Lithocarpus* | PP234613 |
| *L. brevicaudatus* (Skan) Hayata | Fagaceae | *Lithocarpus* | PQ276666 |
| *L. hancei* (Benth.) Rehder | Fagaceae | *Lithocarpus* | MW375417 |
| *L. calophyllus* Chun ex C. C. Huang & Y. T. Chang | Fagaceae | *Lithocarpus* | PP234612 |
| *L. haipinii* Chun | Fagaceae | *Lithocarpus* | SRR16661023 |
| *L. polystachyus* Rehder | Fagaceae | *Lithocarpus* | OL569560 |
| *L. glaber* (Thunb.) Nakai | Fagaceae | *Lithocarpus* | OM388303 |
| *L. longanoides* C. C. Huang & Y. T. Chang | Fagaceae | *Lithocarpus* | SRR16661025 |
| *L. amygdalifolius* (Skan) Hayata | Fagaceae | *Lithocarpus* | PQ276669 |
| *L. tephrocarpus* (Drake) A. Camus | Fagaceae | *Lithocarpus* | SRR16661046 |
| *L. fenzelianus* A. Camus | Fagaceae | *Lithocarpus* | OM388302 |
| *L. dahuensis* H.X.Su, Miao Zhang & B.Hua Chen | Fagaceae | *Lithocarpus* | OP954095 |
| *L. konishii* (Hayata) Hayata | Fagaceae | *Lithocarpus* | PQ276665 |
| *L. uvariifolius* (Hance) Rehder | Fagaceae | *Lithocarpus* | PQ276664 |
| *L. corneus* (Lour.) Rehder | Fagaceae | *Lithocarpus* | PP234614 |
| *L. balansae* (Drake) A. Camus | Fagaceae | *Lithocarpus* | KP299291 |
| *L. tabularis* Y. C. Hsu & H. W. Jen | Fagaceae | *Lithocarpus* | PQ276662 |
| *L. dealbatus* (Hook. f. & Thomson ex Miq.) Rehder | Fagaceae | *Lithocarpus* | PQ276668 |
| *L. cleistocarpus* (Seemen) Rehder & E. H. Wilson | Fagaceae | *Lithocarpus* | OM112296 |
| *L. elizabethiae* (Tutcher) Rehder | Fagaceae | *Lithocarpus* | PQ276667 |
| *L. fenestratus* (Roxb.) Rehder | Fagaceae | *Lithocarpus* | OM112300 |
| *L. grandifolius* (D. Don) S. N. Biswas | Fagaceae | *Lithocarpus* | PQ276659 |
| *L. sphaerocarpus* (Hickel & A. Camus) A. Camus | Fagaceae | *Lithocarpus* | PQ276658 |
| *L. longzhouicus* (C. C. Huang & Y. T. Chang) J. Q. Li & L. Chen | Fagaceae | *Lithocarpus* | PQ276670 |
| *L. areca* (Hickel & A. Camus) A. Camus | Fagaceae | *Lithocarpus* | PQ276671 |
| *L. longipedicellatus* (Hickel & A. Camus) A. Camus | Fagaceae | *Lithocarpus* | PP234611 |
| *L. truncatus* (King ex Hook. f.) Rehder & E. H. Wilson | Fagaceae | *Lithocarpus* | SRR16661105 |
| *L. longinux* (Hu) Chun ex Y.C.Hsu & H.Wei Jen | Fagaceae | *Lithocarpus* | OK181903 |
| *L. pachylepis* A. Camus | Fagaceae | *Lithocarpus* | PQ276660 |
| *L. gymnocarpus* A. Camus | Fagaceae | *Lithocarpus* | PQ276663 |
| *L. fohaiensis* (Hu) A. Camus | Fagaceae | *Lithocarpus* | PQ276656 |
| *L. obscurus* C. C. Huang & Y. T. Chang | Fagaceae | *Lithocarpus* | OM112297 |

**Table S3.** The RSCU values in cp genomes of *L. yongfuensis.*

| AA | Codon | RSCU | AA | Codon | RSCU |
| --- | --- | --- | --- | --- | --- |
| Ala | GCA | 1.08 | Pro | CCA | 1.09 |
| Ala | GCC | 0.63 | Pro | CCC | 0.83 |
| Ala | GCG | 0.49 | Pro | CCG | 0.57 |
| Ala | GCU | 1.8 | Pro | CCU | 1.51 |
| Cys | UGC | 1.46 | Gln | CAA | 1.55 |
| Cys | UGU | 0.54 | Gln | CAG | 0.45 |
| Asp | GAC | 0.39 | Arg | AGA | 1.83 |
| Asp | GAU | 1.61 | Arg | AGG | 0.68 |
| Glu | GAA | 1.53 | Arg | CGA | 1.33 |
| Glu | GAG | 0.47 | Arg | CGC | 0.41 |
| Phe | UUC | 0.68 | Arg | CGG | 0.43 |
| Phe | UUU | 1.32 | Arg | CGU | 1.32 |
| Gly | GGA | 1.52 | Ser | AGC | 0.38 |
| Gly | GGC | 0.47 | Ser | AGU | 1.22 |
| Gly | GGG | 0.7 | Ser | UCA | 1.19 |
| Gly | GGU | 1.31 | Ser | UCC | 1.01 |
| His | CAC | 0.46 | Ser | UCG | 0.54 |
| His | CAU | 1.54 | Ser | UCU | 1.66 |
| Ile | AUA | 0.94 | Thr | ACA | 1.19 |
| Ile | AUC | 0.57 | Thr | ACC | 0.73 |
| Ile | AUU | 1.49 | Thr | ACG | 0.45 |
| Lys | AAA | 1.52 | Thr | ACU | 1.64 |
| Lys | AAG | 0.48 | Val | GUA | 1.54 |
| Leu | CUA | 0.8 | Val | GUC | 0.47 |
| Leu | CUC | 0.37 | Val | GUG | 0.57 |
| Leu | CUG | 0.4 | Val | GUU | 1.43 |
| Leu | CUU | 1.22 | Trp | UGG | 1 |
| Leu | UUA | 1.97 | Tyr | UAC | 0.42 |
| Leu | UUG | 1.24 | Tyr | UAU | 1.58 |
| Asn | AAC | 0.45 | Termination | UAG | 0.53 |
| Asn | AAU | 1.55 | Termination | UGA | 0.65 |
| Met | AUG | 1.0 | Termination | UAA | 1.82 |

**Table S4.** Nucleotide polymorphism analysis of 35 *Lithocarpus* chloroplast genomes.

| **gene** | **Pi** | **IGS** | | **Pi** |
| --- | --- | --- | --- | --- |
| *trnH-GUG* | 0.00757 | | *trnH-GUG-psbA* | 0.00756 |
| *psbA* | 0.00121 | | *psbA-trnK-UUU_2* | 0.00317 |
| *trnK-UUU* | 0.00828 | | *trnK-UUU_2-matK* | 0.00129 |
| *matK* | 0.00615 | | *matK-trnK-UUU_1* | 0.00699 |
| *rps16* | 0.00207 | | *trnK-UUU_1-rps16_2* | 0.01408 |
| *trnQ-UUG* | 0.00079 | | *rps16_2-rps16_1* | 0.0042 |
| *psbK* | 0.00121 | | *rps16_1-trnQ-UUG* | 0.00506 |
| *psbI* | 0.00351 | | *trnQ-UUG-psbK* | 0.00467 |
| *trnS-GCU* | 0 | | *psbK-psbI* | 0.01074 |
| *trnG-GCC* | 0.01479 | | *psbI-trnS-GCU* | 0.00251 |
| *trnR-UCU* | 0.0008 | | *trnR-UCU-atpA* | 0.01267 |
| *atpA* | 0.00129 | | *atpA-atpF_2* | 0.0019 |
| *atpF* | 0.00173 | | *atpF_2-atpF_1* | 0.00364 |
| *atpH* | 0.00253 | | *atpF_1-atpH* | 0.00923 |
| *atpI* | 0.00114 | | *atpH-atpI* | 0.00379 |
| *rps2* | 0.00231 | | *atpI-rps2* | 0.00261 |
| *rpoC2* | 0.00234 | | *rps2-rpoC2* | 0.00332 |
| *rpoC1* | 0.00206 | | *rpoC2-rpoC1_2* | 0.00166 |
| *rpoB* | 0.00299 | | *rpoC1_2-rpoC1_1* | 0.00523 |
| *trnC-GCA* | 0.00901 | | *rpoC1_1-rpoB* | 0 |
| *petN* | 0.00187 | | *rpoB-trnC-GCA* | 0.00553 |
| *psbM* | 0 | | *trnC-GCA-petN* | 0.00488 |
| *trnD-GUC* | 0 | | *petN-psbM* | 0.0035 |
| *trnY-GUA* | 0 | | *psbM-trnD-GUC* | 0.00461 |
| *trnE-UUC* | 0 | | *trnD-GUC-trnY-GUA* | 0.01062 |
| *trnT-GGU* | 0.08282 | | *trnY-GUA-trnE-UUC* | 0.00273 |
| *psbD* | 0.00154 | | *trnE-UUC-trnT-GGU* | 0.00503 |
| *psbC* | 0.00172 | | *trnT-GGU-psbD* | 0.00509 |
| *trnS-UGA* | 0.00061 | | *psbC-trnS-UGA* | 0.01243 |
| *rps14* | 0.00168 | | *rps14-psaB* | 0.00172 |
| *psaB* | 0.00146 | | *psaB-psaA* | 0 |
| *psaA* | 0.00098 | | *psaA-ycf3_3* | 0.00367 |
| *ycf3* | 0.00056 | | *ycf3_3-ycf3_2* | 0.00612 |
| *rps4* | 0.0018 | | *ycf3_2-ycf3_1* | 0.00451 |
| *trnT-UGU* | 0.00078 | | *rps4-trnT-UGU* | 0.00596 |
| *trnL-UAA* | 0 | | *trnT-UGU-trnL-UAA_1* | 0.00385 |
| *trnF-GAA* | 0.00078 | | *trnL-UAA_1-trnL-UAA_2* | 0.00199 |
| *ndhJ* | 0.00036 | | *trnL-UAA_2-trnF-GAA* | 0.00574 |
| *ndhK* | 0.00222 | | *trnF-GAA-ndhJ* | 0.00907 |
| *ndhC* | 0.0024 | | *ndhJ-ndhK* | 0.02438 |
| *trnV-UAC* | 0 | | *ndhK-ndhC* | 0.00181 |
| *atpE* | 0.00252 | | *ndhC-trnV-UAC_2* | 0.00671 |
| *atpB* | 0.00249 | | *trnV-UAC_2-trnV-UAC_1* | 0.00425 |
| *rbcL* | 0.00206 | | *atpB-rbcL* | 0.0043 |
| *accD* | 0.002 | | *rbcL-accD* | 0.01976 |
| *psaI* | 0 | | *accD-psaI* | 0.00319 |
| *ycf4* | 0.002 | | *psaI-ycf4* | 0.003 |
| *cemA* | 0.00252 | | *ycf4-cemA* | 0.00307 |
| *petA* | 0.00406 | | *cemA-petA* | 0.00365 |
| *psbJ* | 0 | | *petA-psbJ* | 0.00494 |
| *psbL* | 0 | | *psbJ-psbL* | 0.00244 |
| *psbF* | 0.00143 | | *psbL-psbF* | 0 |
| *psbE* | 0 | | *psbF-psbE* | 0.00635 |
| *petL* | 0.0006 | | *psbE-petL* | 0.00661 |
| *petG* | 0 | | *petL-petG* | 0.00751 |
| *trnW-CCA* | 0 | | *petG-trnW-CCA* | 0.00172 |
| *psaJ* | 0.00042 | | *psaJ-rpl33* | 0.0069 |
| *rpl33* | 0.00211 | | *rpl33-rps18* | 0.00216 |
| *rps18* | 0.00234 | | *rps18-rpl20* | 0.00366 |
| *rpl20* | 0.00485 | | *rpl20-rps12_1* | 0.00654 |
| *rps12* | 0 | | *rps12_1-clpP_3* | 0.0021 |
| *clpP* | 0.00212 | | *clpP_3-clpP_2* | 0.0045 |
| *psbB* | 0.0012 | | *clpP_2-clpP_1* | 0.00482 |
| *psbT* | 0.00053 | | *clpP_1-psbB* | 0.00589 |
| *psbN* | 0.00084 | | *psbB-psbT* | 0.01022 |
| *psbH* | 0.00051 | | *psbT-psbN* | 0 |
| *petB* | 0.00106 | | *psbN-psbH* | 0.0021 |
| *rpoA* | 0.00311 | | *psbH-petB_1* | 0.00619 |
| *rps11* | 0.00436 | | *petB_1-petB_2* | 0.00211 |
| *rpl36* | 0.00251 | | *rpoA-rps11* | 0 |
| *infA* | 0.00796 | | *rps11-rpl36* | 0.00312 |
| *rps8* | 0.00324 | | *rpl36-infA* | 0.01403 |
| *rpl14* | 0.00214 | | *infA-rps8* | 0.00366 |
| *rpl16* | 0.00083 | | *rps8-rpl14* | 0.00163 |
| *rps3* | 0.00295 | | *rpl14-rpl16_2* | 0.00164 |
| *rpl22* | 0.00875 | | *rpl16_2-rpl16_1* | 0.00331 |
| *rps19* | 0.00189 | | *rpl16_1-rps3* | 0.00314 |
| *rpl2* | 0.00033 | | *rps3-rpl22* | 0.00662 |
| *rpl23* | 0 | | *rpl22-rps19* | 0.00429 |
| *ycf2* | 0.00035 | | *rps19-rpl2_2* | 0.00504 |
| *trnL-CAA* | 0.00141 | | *rpl2_2-rpl2_1* | 0.00037 |
| *ndhB* | 0.00007 | | *rpl2_1-rpl23* | 0 |
| *rps7* | 0 | | *ycf2-trnL-CAA* | 0.00053 |
| *trnV-GAC* | 0 | | *trnL-CAA-ndhB_2* | 0.0001 |
| *trnA-UGC* | 0.00078 | | *ndhB_2-ndhB_1* | 0.00221 |
| *trnR-ACG* | 0 | | *ndhB_1-rps7* | 0 |
| *trnN-GUU* | 0 | | *rps7-rps12_3* | 0 |
| *ycf1* | 0.00767 | | *rps12_3-rps12_2* | 0.00021 |
| *ndhF* | 0.00668 | | *rps12_2-trnV-GAC* | 0.00077 |
| *rpl32* | 0.00765 | | *trnA-UGC_1-trnA-UGC_2* | 0.00021 |
| *trnL-UAG* | 0 | | *trnR-ACG-trnN-GUU* | 0.00581 |
| *ccsA* | 0.00175 | | *trnN-GUU-ycf1* | 0.00085 |
| *ndhD* | 0.00415 | | *ndhF-rpl32* | 0.00559 |
| *psaC* | 0.00116 | | *rpl32-trnL-UAG* | 0.00781 |
| *ndhE* | 0.00075 | | *trnL-UAG-ccsA* | 0.0012 |
| *ndhG* | 0.00408 | | *ccsA-ndhD* | 0.01823 |
| *ndhI* | 0.00427 | | *ndhD-psaC* | 0.0074 |
| *ndhA* | 0.00506 | | *psaC-ndhE* | 0.00448 |
| *ndhH* | 0.00255 | | *ndhE-ndhG* | 0.00947 |
| *rps15* | 0.00407 | | *ndhG-ndhI* | 0.00435 |
|  |  | | *ndhI-ndhA_2* | 0.00205 |
|  |  | | *ndhA_2-ndhA_1* | 0.00636 |
|  |  | | *ndhA_1-ndhH* | 0 |
|  |  | | *ndhH-rps15* | 0.00881 |
|  |  | | *rps15-ycf1* | 0.00813 |
|  |  | | *ycf1-trnN-GUU* | 0.00035 |
|  |  | | *trnN-GUU-trnR-ACG* | 0.00581 |
|  |  | | *trnA-UGC_2-trnA-UGC_1* | 0.00021 |
|  |  | | *trnV-GAC-rps12_2* | 0.00077 |
|  |  | | *rps12_2-rps12_3* | 0.00021 |
|  |  | | *rps12_3-rps7* | 0 |
|  |  | | *rps7-ndhB_1* | 0 |
|  |  | | *ndhB_1-ndhB_2* | 0.00213 |
|  |  | | *ndhB_2-trnL-CAA* | 0.0001 |
|  |  | | *trnL-CAA-ycf2* | 0.00053 |
|  |  | | *rpl23-rpl2_1* | 0 |
|  |  | | *rpl2_1-rpl2_2* | 0.00034 |
|  |  | | *rpl2_2-trnH-GUG* | 0.00847 |
